# Altered Memory T-Cell Responses to Bacillus Calmette-Guerin and Tetanus Toxoid Vaccination and Altered Cytokine Responses to Polyclonal Stimulation in HIV-Exposed Uninfected Kenyan Infants

**DOI:** 10.1371/journal.pone.0143043

**Published:** 2015-11-16

**Authors:** Miguel A. Garcia-Knight, Eunice Nduati, Amin S. Hassan, Faith Gambo, Dennis Odera, Timothy J. Etyang, Nassim J. Hajj, James Alexander Berkley, Britta C. Urban, Sarah L. Rowland-Jones

**Affiliations:** 1 NDM Research Building, Nuffield Department of Clinical Medicine, University of Oxford, Oxford, United Kingdom; 2 KEMRI-Wellcome Trust Research Program, Centre for Geographical Medicine Research, Kilifi, Kenya; 3 Comprehensive Care and Research Clinic, Kilifi County Hospital, Kilifi, Kenya; 4 Department of Parasitology, Liverpool School of Tropical Medicine, Pembroke Place, Liverpool, United Kingdom; University British Columbia, CANADA

## Abstract

Implementation of successful prevention of mother-to-child transmission of HIV strategies has resulted in an increased population of HIV-exposed uninfected (HEU) infants. HEU infants have higher rates of morbidity and mortality than HIV-unexposed (HU) infants. Numerous factors may contribute to poor health in HEU infants including immunological alterations. The present study assessed T-cell phenotype and function in HEU infants with a focus on memory Th1 responses to vaccination. We compared cross-sectionally selected parameters at 3 and 12 months of age in HIV-exposed (n = 42) and HU (n = 28) Kenyan infants. We measured *ex vivo* activated and bulk memory CD4 and CD8 T-cells and regulatory T-cells by flow cytometry. In addition, we measured the magnitude, quality and memory phenotype of antigen-specific T-cell responses to Bacillus Calmette-Guerin and Tetanus Toxoid vaccine antigens, and the magnitude and quality of the T cell response following polyclonal stimulation with staphylococcal enterotoxin B. Finally, the influence of maternal disease markers on the immunological parameters measured was assessed in HEU infants. Few perturbations were detected in *ex vivo* T-cell subsets, though amongst HEU infants maternal HIV viral load positively correlated with CD8 T cell immune activation at 12 months. Conversely, we observed age-dependent differences in the magnitude and polyfunctionality of IL-2 and TNF-α responses to vaccine antigens particularly in Th1 cells. These changes mirrored those seen following polyclonal stimulation, where at 3 months, cytokine responses were higher in HEU infants compared to HU infants, and at 12 months, HEU infant cytokine responses were consistently lower than those seen in HU infants. Finally, reduced effector memory Th1 responses to vaccine antigens were observed in HEU infants at 3 and 12 months and higher central memory Th1 responses to *M*. *tuberculosis* antigens were observed at 3 months only. Long-term monitoring of vaccine efficacy and T-cell immunity in this vulnerable population is warranted.

## Introduction

Due to the success of antiretroviral therapy (ART) strategies, mother-to-child transmission of HIV-1 has been virtually eliminated in countries with universal access to health care and is in steep decline in many sub-Saharan African countries[1,2]. However, it has been frequently reported that infants born to HIV-1-infected mothers who are themselves free from HIV infection, termed HIV-exposed uninfected (HEU) infants, suffer from higher rates of infectious diseases[3,4] and mortality[5] than infants born to HIV uninfected mothers. Currently in some areas of southern Africa, up to 30% of all new-borns are HEU[6]. Thus, defining factors that underlie poor health in HEU infants is a public health priority for countries with high HIV-1 prevalence[2].

Being born into an HIV-infected household has clear implications regarding infant care. Mothers may themselves be ill or suffering economic consequences of their infection. Infant feeding practices may also be sub-optimal. Importantly, HEU infants may also experience heightened vertical exposure to maternal co-infections such as cytomegalovirus (CMV) and *Mycobacterium tuberculosis*[7]. In addition, *in utero* exposure to antiretroviral drugs and maternal immune system perturbations, including inflammation from HIV-1 infection during foetal and/or neonatal development may have lasting effects on infant immunity.

Immune abnormalities such as reduced maternal antibody transfer to the foetus have been described in HEU infants[8,9]. Maternal antibodies are critical in mediating early infant immunity and reduced levels probably contribute to infection susceptibility. Sustained subclinical reductions in lymphocytes, neutrophils and platelets[10,11] persisting into late childhood[12] have also been described. In addition, phenotypic alterations, particularly in T-cells, indicate altered memory subset distribution and heightened immune activation[13,14]. HIV-1-specfic T-cell responses in HEU neonates[15,16] under the influence of regulatory T-cells (Tregs)[17] have also been reported, suggesting *in utero* priming of T-cells by HIV-1 antigens. The functional consequences of these alterations are unclear; to address this a common approach has been to measure responses to routine immunisation, a scenario of a controlled antigenic challenge.

Most infant vaccinations elicit protection through neutralizing antibody induction. There are conflicting reports regarding antibody induction in HEU infants with reports of higher[8] or lower[18] antibody titres. Reports on vaccine-specific T-cell responses are also conflicting (reviewed in[7]). Most studies have focused on Bacillus Calmette-Guerin (BCG), the only vaccine under the Expanded Programme for Immunization thought to mediate protection directly through T-cells. Due to the increasing number of HEU infants born in regions with twin HIV/tuberculosis epidemics, assessing BCG responses and the induction of vaccine-specific memory has important public health implications.

Here we cross-sectionally compared T-cell phenotype and function in Kenyan HEU and HIV-unexposed (HU) infants at 3 and 12 months of age. We assessed the frequencies of *ex vivo* activated T-cells, Tregs and memory T-cell subsets. In addition, we measured single and polyfunctional cytokine responses following vaccination with BCG and TT delivered under the Kenyan Expanded programme for Immunisation and following polyclonal stimulation. Our principal aim was to examine the memory phenotype of cytokine-responsive cells to vaccine antigens. Finally, we assessed the role of maternal disease markers in modulating T cell immunity in HEU infants.

## Methods

### Participants

The study was conducted between 2011 and 2013 and nested within a longitudinal cohort of HEU infants recruited to assess adaptive immunity following exposure to HIV[19]. The protocol was approved by the Kenyan Medical Research Institute Ethical Research Council (protocol no. SSC 2085) and the Oxford Tropical Research Ethics Committee (ref: 45–11); written informed consent was obtained from mothers or guardians. Infants born to HIV-1 infected women (N = 42) were recruited from the Comprehensive Care and Research Clinic (CCRC), Kilifi County Hospital, Coastal Province, Kenya. PMTCT followed national guidelines[20] and WHO recommendations[21] and included cotrimoxazole prophylaxis from six weeks to 18 months of age in HIV-exposed infants. Pregnant women were given time-limited prophylactic ART for PMTCT of HIV-1 if their CD4 count was >350 CD4 T cells/uL (azidothymidine [AZT] from 14 weeks of pregnancy or at first contact with antenatal services if later, and continued AZT prophylaxis through labour and one week after delivery) or put on lifelong highly active antiretroviral therapy (HAART) if their CD4 count was< 350 cell/μL. HIV exposed infants born to mothers not on HAART were prescribed nevirapine prophylaxis at birth to be continued until one week after complete cessation of breastfeeding; infants born to mothers on HAART were prescribed nevirapine prophylaxis up to six weeks of life. Mothers were counselled on safe breastfeeding practices HIV-1 status was determined for all infants attending the CCRC by PCR at six weeks of age or at first contact and by rapid antibody test at 9 and 18 months of age. A 15 mL blood sample was obtained from the mothers of participating infants from the CCRC. Community control HU infants (N = 28) were recruited from three localities within the catchment area of Kilifi County and sampled at a single time point. Due to ethical considerations, HU infants were not directly screened for HIV infection. N = 16 infants classed as HU (all 3 month old infants and six 12 month old infants) were born to HIV-negative mothers tested antenatally. Antenatal screening data was not available for the remaining n = 12 infants classed as HU, who were recruited as part of an annual malaria epidemiological survey. HIV prevalence in Coastal Province in women aged 25–35 years is 4.1%[22]. The severity of HIV-1 pathology in early infancy and low maternal prevalence relative to the national average in women (7.6%[23]) made the recruitment of HIV-1 positive or HEU infants unlikely. Vaccination was in accordance with the Kenyan Expanded Programme for Immunisation which includes BCG (BCG-Russia, Serum Institute of India, Pune, Maharashtra, India) at birth and Diphtheria/Tetanus/Pertussis/Hepatitis B/Haemophilus influenza type B (Serum Institute of India) pentavalent vaccine at 6, 10 and 14 weeks of age. Vaccination data were obtained from infant vaccination cards. 5mL infant whole-blood samples for the present study were collected, stored at room temperature and processed within 4 hours. Complete blood counts were determined using a Coulter MDII-18 counter (Beckman-Coulter, Fullerton CA, USA). Summary data on maternal ART usage and CD4 counts are presented elsewhere[19] and were obtained from clinical records.

### Maternal viral load determination

Maternal viral loads were determined at the point of infant recruitment. This was done at the International Centre for Reproductive Health, Mombasa Kenya, using a RT-qPCR test developed by the Agence Nationale de Recherches sur le SIDA (ANRS)[24]. The assay targets a conserved long terminal repeat region and has a detection limit of 300 RNA copies/ml.

### Immunophenotyping

Plasma was removed from whole-blood and replaced with RPMI 1640 medium plus 10% inactivated new-born calf serum, 0.01% β-mercaptoethanol, 1% penicillin/streptomycin, 1% L-glutamate and 1% Hepes (R10). 100μL of resuspended cells were incubated with fluorescently-labelled antibodies (S1 Table) following standard protocols[25]; red blood cells (RBCs) were lysed (FACS lysing solution; BD Bioscience, San Jose, CA) and washed in phosphate buffered saline (PBS). To detect intracellular Bcl-2, surface-stained cells were permeabilised (permeabilisation buffer, BD Bioscience). The Human Regulatory T-cell Whole-Blood staining kit (eBiosciences, San Diego, CA) was used according to the manufacturer’s instructions to detect Tregs. Following permeabilisation, cells were blocked with 2% rat serum and incubated with anti-FOXP3. Isotype controls processed similarly were used to set positive gates for markers except CD3, CD4, CD8 and CD25.

### Whole-blood stimulation and intracellular cytokine staining

A short-term stimulation assay for intracellular cytokine staining (ICS) in whole blood was adapted [26]. Briefly, plasma was replaced with R10; 200μL of blood cells in R10 were cultured with CD49d and CD28 alone (0.5ug/mL each; eBiosciences; unstimulated control), or in combination with either TT (Indian Serum Institute, Pune, India; 10ug/mL), purified protein derivative (PPD) of *M*. *tuberculosis* (Statens Serum Institute, Copenhagen, Denmark; 20ug/mL) or staphylococcal enterotoxin B (SEB; Sigma-Aldrich, St. Louis, Missouri; 1ug/mL). After 7 hours in a water bath at 37°C, brefeldin A (Sigma-Aldrich) was added (10ug/mL). After 5 hours the heat switched off. After 10hrs, RBCs were lysed and white cells fixed (FACS lysing solution; BD Bioscience) and cryopreserved. Batched stimulated cells were thawed, washed in PBS and permeabilised. Fluorescently-labelled antibodies were added (S1 Table); following incubation, cells were washed before acquisition.

### Flow cytometry

A Cyan ADP (Beckman Coulter, Pasadena, CA) flow cytometer was used for acquisition. Compensation controls using anti-mouse κ beads (BD Bioscience) were used for all experiments. For *ex vivo* phenotyping, >70,000 CD3^+^ events were collected. For all ICS experiments all cells were acquired, resulting in acquisition of a mean 4.09 x10^5^ (standard deviation [SD] 2.43 x10^5^) total events and 2.20x10^5^ (SD, 1.15x10^5^) CD3^+^ events. Following vaccine antigen stimulation, a mean of 712 (SD 544) events in the cytokine^+^ gates were recorded.

### Data analysis

Compensation and gating was done in FlowJo v7.6 (Treestar, Ashland, Oregon). Statistics were done in Prism v6 (GraphPad Software, La Jolla, CA). Differences between independent groups were assessed by an unpaired t-test for normally distributed data or by a Mann-Whitney U test for data not normally distributed. *χ* ^*2*^ was used to compare categorical data. Approximate absolute CD3 T cell counts were obtained by dividing the absolute lymphocyte count by 100 and multiplying the result by the frequency of CD3 T cells obtained from the flow cytometry lymphocyte gate. Approximate absolute CD4 and CD8 T cell counts were similarly calculated based on the approximate CD3 T cell count and the frequencies of CD4 and CD8 T cells relative to the flow cytometry parent gate. A *P* value ≤0.05 was considered significant. For ICS analysis, positive events in the unstimulated controls were subtracted from PPD, TT or SEB stimulations. Responses were considered positive for responder frequency analysis (S3 Fig) if adjusted values were ≥0.01% of total CD4 T-cells. Negative values, resulting from measurement errors, were set to zero; to compensate for this potential systematic bias, positive values <0.01% were set to zero as detailed in [27]. Boolean gating was used to assess frequencies of cells expressing cytokine combinations; the data was processed in Excel and Spice version 5.3[27]. Exclusion from ICS analysis occurred if a) vaccination was not recorded or the relevant doses were not received (vaccine schedule failure); b) insufficient sample was available or 3) the adjusted positive control (SEB) value was < the median plus 3 median absolute deviations of the unstimulated samples for all infants (failed positive control). Analysis of TT responses at 3 months was done in infants who received 2 pentavalent vaccine doses or who received the 3^rd^ <72hrs of sampling. This cut off was set based on Cellerai et al. where equivalent Th1 CD4 T cell responses to TT stimulation were shown up to 72hrs post revaccination in adults compared to baseline [28]. TT responses at 12 months were analysed in infants who had received all 3 pentavalent vaccine doses.

## Results

### Infant characteristics and sample exclusion

81 specimens from 65 infants were analysed (S1 Fig). Age and gender were well matched between HU and HEU infants within age groups (Table 1). *Ex vivo* analyses were done on all specimens from 3 month-old infants and in a subset (n = 32) of specimens from 12 month-old infants. For antigen-specific analyses, after applying exclusion criteria, 10, 22 and 6 specimens were excluded for the PPD, TT and SEB analyses, respectively (Table 1).

**Table 1:** Infant age, gender, timing of relevant vaccinations and sample exclusions.

Characteristic	Month 3		*P*	Month 12		*P*
	HU (10)	HEU (19)		HU (18)	HEU (34)	
Median age, months (range)	3.5 (2.5–4.8)	4.0 (2.6–5.6)	NS	12.3 (9.6–13.9)	12.1 (10.7–14.0)	NS
Female sex (%)	6 (60)	8 (42)	NS	12 (67)	17 (50)	NS
Median age at BCG, days (IQR)	5 (1.5–12.5)	3.5 (1–12.5)	NS	4 (2–23)	9 (1–34)	NS
Median age at 1^st^ PV, weeks (IQR)	6.5 (6.2–7.0)	6.2 (6.0–6.9)	NS	6.3 (6.1–6.6)	6.3 (6.0–7.4)	NS
1^st^ PV, median days off schedule (IQR)	4 (1–7)	2 (0–6)	NS	2 (1–7)	2.0 (1–10)	NS
Median age at 2^nd^ PV, weeks (IQR)	11 (10.6–11.6)	10.2 (10.0–11.8)	NS	10.9 (10.6–12.9)	10.9 (10.1–12.7)	NS
2^nd^PV, median days off schedule (IQR)	7 (4–11)	2 (0–12)	NS	9 (4–23)	6.5 (1–19)	NS
Median age at 3^rd^ PV, weeks (IQR)	NA	NA	-	15.4 (14.8–29.6)	15.8 (14.3–18.9)	NS
3^rd^PV, median days off schedule (IQR)	NA	NA	-	10 (7–26)	12.5 (2–34)	NS
N infants excluded from PPD analysis	1^c^	1^a^, 2^b^	-	1^c^	2^a^, 1^b^, 2^c^	-
N infants excluded from TT analysis	2^a^	11^a^	-	1^c^	5^a^, 2^b^,1^c^	-
N infants excluded from SEB analysis	1^c^	2^b^	-	1^c^	2^c^	-

An unpaired t-test was used to compare continuous parameters; *χ* ^*2*^ was to compare categorical data; NS, not significant; NA not applicable; PV pentavalent vaccination; HEU, HIV exposed uninfected; HU, HIV-unexposed; IQR, inter-quartile range

^a^ Vaccine schedule failure;

^b^ insufficient sample volume;

^c^ failed positive control.

### Comparable *ex vivo* frequencies of CD4 and CD8 T-cell subsets between HEU and HU infants at 3 and 12 months of age

Previous reports have shown decreased CD4 and CD8 T cell absolute counts in HEU infants compared to controls that have been attributed to exposure to ART[10,11,29]. In addition, increased T-cell frequencies with antigen-experienced[13,14] and regulatory[17] phenotypes have been reported in HEU infants.

Analysis of absolute CD3 and CD8 T cell counts in our data indicated a significant increase in these parameters at 12 months in HEU infants but not at 3 months (S2 Table). Although a trend towards an increased absolute number of CD4 T cells in HEU infants was also observed at 12 months, no significant differences were observed in CD4 and CD8 T cell percentages or in the CD4/CD8 T cell ratio at either time point (S2 Table).

We analysed frequencies of activated T-cells and Tregs at 3 and 12 months of age and bulk memory subsets at 12 months. Surprisingly, activated and PD-1-expressing CD4 T-cell frequencies were significantly higher in HU infants at 3 months of age and activated CD4 T-cell frequencies remained higher at 12 months (Table 2). We found no significant differences in expression levels of any marker of CD8 T-cell activation or exhaustion (S3 Table). Similarly, no significant changes in circulating Treg frequencies between HU and HEU infants at 3 and 12 months were found (Table 2). To assess memory subsets, CD45RA and CCR7[30], as well as the homeostatic maintenance marker CD127[31] and the anti-apoptotic marker Bcl-2[32], were used. No significant redistributions of memory subsets were observed in CD4 or CD8 T-cells in HEU infants or in expression levels of CD127 and Bcl-2 (Table 2 and S3 Table). A trend towards a reduction in bulk effector memory CD4 T cells was observed in HEU infants at 12 months of age (Table 2).

**Table 2:** *Ex vivo* CD4 T cell immune activation, Tregs and memory cell phenotypes.

Characteristic	3 months median % of CD4 T cells (range)			12 months median % of CD4 T cells (range)		
	HU (n = 10)	HEU (n = 19)	^¥^ *P*	HU (n = 16)	^Φ^HEU (n = 16)	*P*
**Activation & exhaustion**						
CD38^+^ HLA-DR^+^	6.8 (3.0–13.7)	3.2 (2.0–12.8)	0.01	6.2 (1.7–14.0)	2.7 (1.4–11.9)	0.04
PD-1	12.9 (8.7–22.9)	8.7 (1.1–16.8)	0.01	13.6 (7.2–25.1)	11.5 (8.8–26.1)	NS
Tim-3	3.0 (1.2–5.0)	2.1 (0.3–10.0)	NS	1.8 (0.5–3.7)	2.4 (1.3–9.7)	NS
**Regulation**						
CD25^hi^ FoxP3^+^	5.1 (1.4–7.0)	4.6 (1.7–6.8)	NS	4.8 (1.81–7.96)	3.9 (1.2–7.7)	NS
**Anti-apoptosis**						
Bcl-2^+^	-	-	NA	74.55 (22.2–92.2)	72.1 (47.9–89.6)	NS
Bcl-2^-^	-	-	NA	25.45 (7.8–77.8)	30.6 (10.4–55.0)	NS
**Memory**						
CD127^+^	-	-	NA	84.5 (69.8–93.4)	83.7 (74.8–94.9)	NS
CD127^-^	-	-	NA	16.2 (6.6–30.6)	16.9 (5.4–26.4)	NS
Naïve	-	-	NA	66.2 (43.5–83.9)	73.5 (19.2–83.0)	NS
T_CM_	-	-	NA	16.15 (8.5–42.8)	14.0 (4.5–19.3)	NS
T_EMRA_	-	-	NA	5.6 (0.7–10.8)	2.6 (1.0–52.2)	NS
T_EM_	-	-	NA	10.2 (4.0–22.5)	6.9 (2.8–24.1)	0.06

^¥^ *P* values were calculated using the Mann-Whitney U test.

^Φ^ CD127 expression was measured on n = 11 samples.

NS = not significant NA = not applicable; HEU, HIV-exposed uninfected; HU, HIV-unexposed

### Altered mono and polyfunctional cytokine responses to stimulation with PPD, TT and SEB in HEU relative to HU infants

We assessed the magnitude and polyfunctionality of CD4 and CD8 T-cell cytokine responses (IFN-γ, IL-2 and TNF-α) to vaccine antigens and polyclonal stimulation (S2A–S2E Fig). Similar frequencies of HEU and HU infant responders to both vaccine antigens at both time points was found (S3A and S3B Fig).

**Fig 1:**
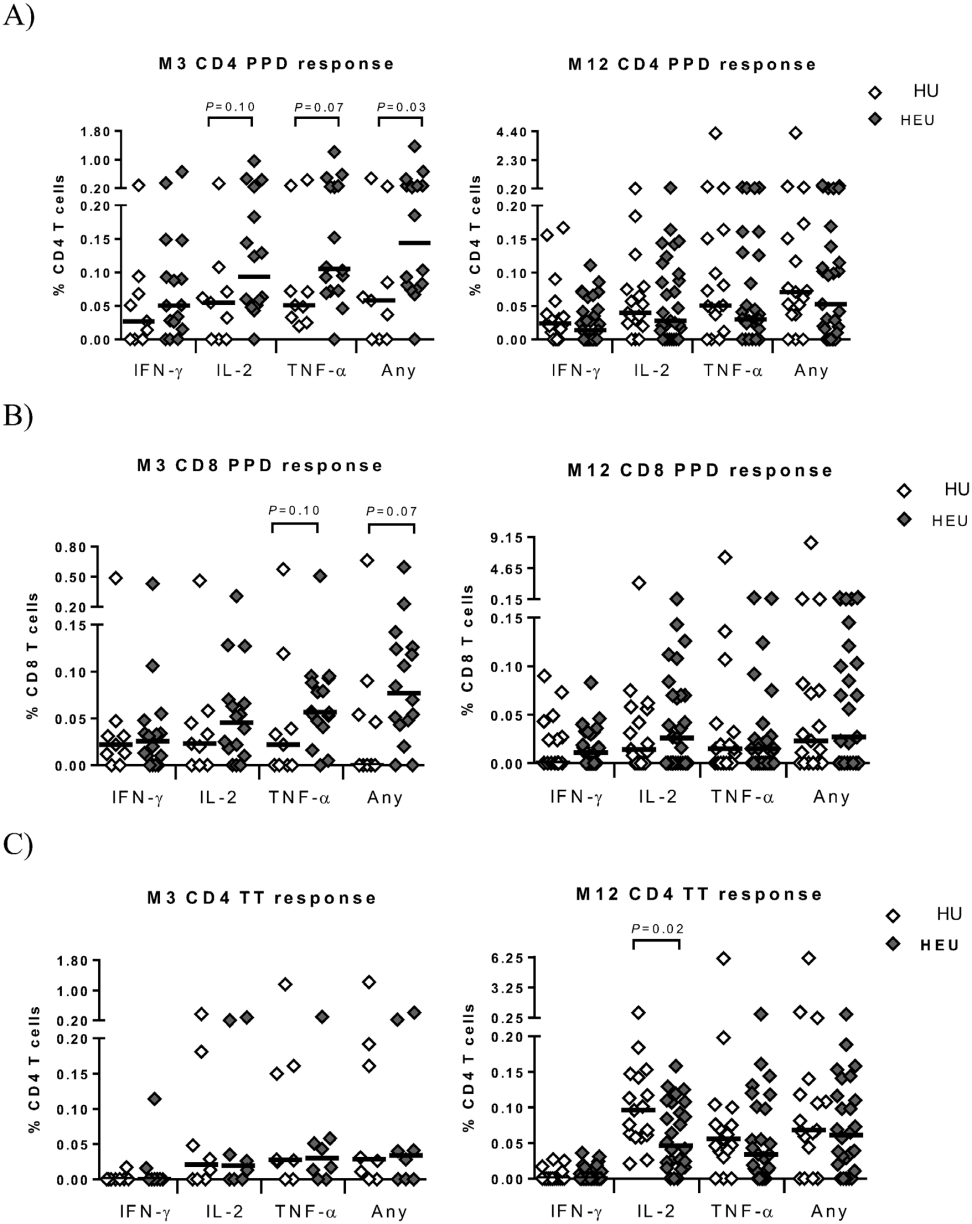
Single cytokine CD4 and CD8 T-cell responses to vaccine antigens. The magnitude of CD4 (A) and CD8 (B) T-cell IFN-γ, IL-2, TNF-α or any cytokine (Any) responses following short term stimulation with PPD in HIV-unexposed (HU) and HIV-exposed uninfected (HEU) infants in month 3 (M3) and month 12 (M12) age groups. Similarly, the magnitude of cytokine responses to TT are shown in (C) for CD4 T-cells only. The horizontal black line is the median frequency of T-cells expressing the indicated cytokine or any cytokine. The Mann Whitney U test was used to assess differences between the two groups.

**Fig 2:**
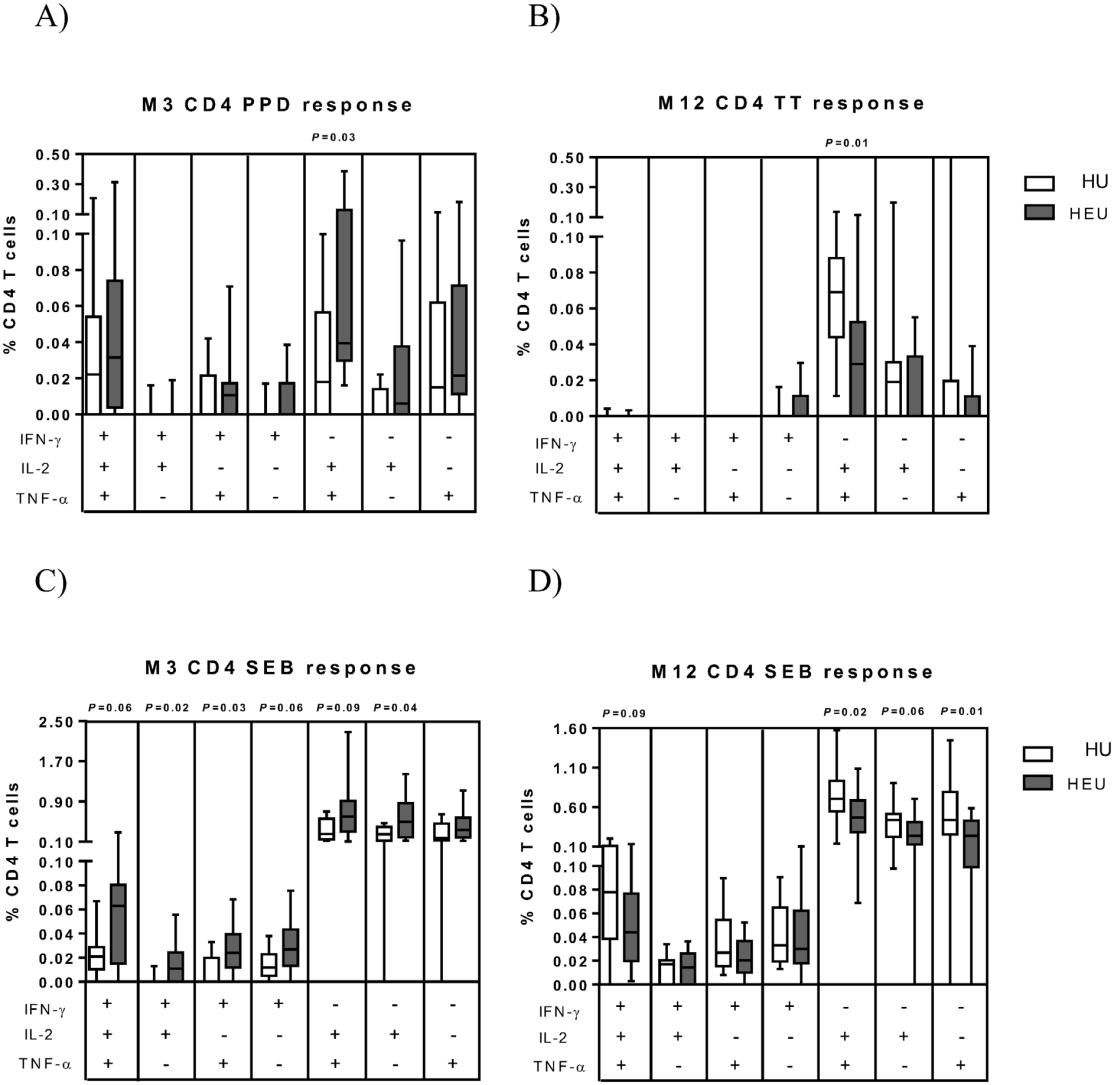
Polyfunctional CD4 T-cell responses to stimulation with vaccine antigens and following polyclonal stimulation with SEB. (A) CD4 T-cells expressing combinations of IFN-γ, IL-2 and/or TNF-α were analysed using Boolean gating following short-term stimulation with PPD in HIV-unexposed (HU) and HIV-exposed uninfected (HEU) infants in the month 3 (M3) age group. (B) A similar analysis is shown assessing TT responses in the month 12 (M12) age group. Similarly, polyfunctional SEB responses in M3 (C) and M12 (D) age groups are shown. The black line is the median frequency of T cells expressing the indicated cytokine combination, the box is the interquartile range and the whiskers the 10^th^ and 90^th^ percentiles. The Mann Whitney U test was used to assess differences between the two groups.

Th1 responses to BCG vaccination at birth peak at 6–10 weeks of age [33]. At 3 months, the time-point closest to peak response, a significantly higher bulk response with any cytokine to PPD as well as a trend towards higher IL-2 and TNF-α responses was observed in HEU infants (Fig 1A); this was mirrored by a significantly higher dual IL-2/TNF-α response at this time point following polyfunctional analysis (Fig 2A). At 12 months, no differences were apparent in bulk Th1 cytokine responses (Fig 1A) or polyfunctionality (S4A Fig). In CD8 T-cells, a trend to a higher TNF-α and any cytokine response was observed in HEU infants at 3 months; no differences were apparent at 12 months (Fig 1B) or in polyfunctional responses at either time-point (S5A Fig).

Following TT stimulation, no differences were observed in magnitude of Th1 cytokine responses (Fig 1C) or polyfunctionality (S4B Fig) between HU and HEU infants at 3 months However, at 12 months a significant decrease in the magnitude of the IL-2 response (Fig 1C) as well as the polyfunctional IL-2/TNF-α response (Fig 2B) was observed in HEU infants.

**Fig 3:**
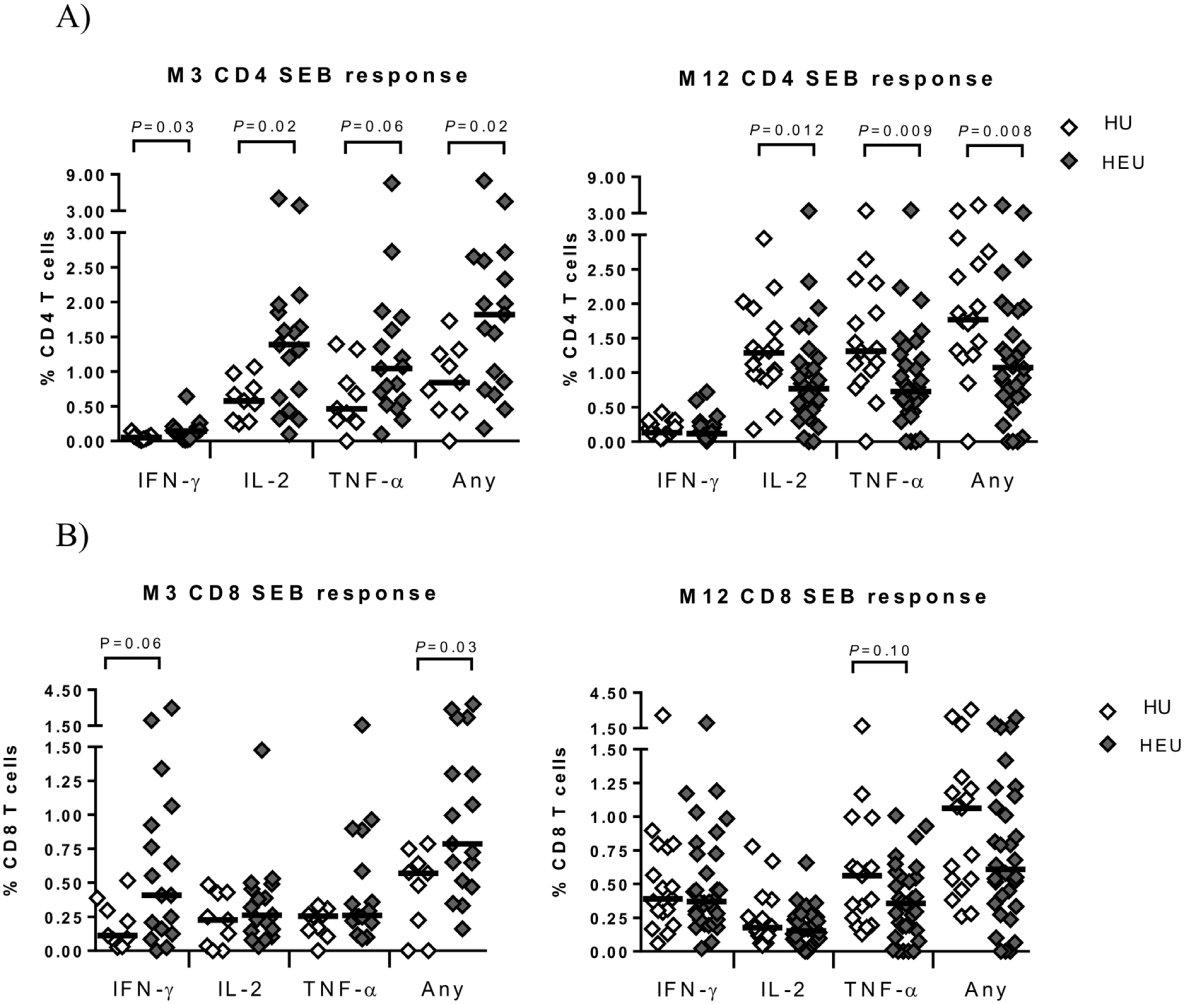
Single cytokine CD4 and CD8 T-cell responses to polyclonal stimulation with SEB. The magnitude of CD4 (A) and CD8 (B) T-cell IFN-γ, IL-2, TNF-α or any cytokine (Any) responses following short-term stimulation with SEB in HIV-unexposed (HU) and HIV- exposed uninfected (HEU) infants in month 3 (M3) and month 12 (M12) age groups. The black line is the median frequency of T-cells expressing the indicated cytokine or any cytokine. The Mann Whitney U test was used to assess differences between the two groups.

Following SEB stimulation, significantly higher CD4 T-cell expression of IFN-γ, IL-2 and any cytokine, as well as a trend to higher TNF-α expression, was observed in HEU compared to HU infants at 3 months following analysis of single cytokine responses (Fig 3A); this was mirrored, following polyfunctional analysis, by significantly higher dual IFN-γ/IL-2 and IFN-γ/TNF-α and single IL-2-expression as well as trends towards higher triple IFN-γ/IL-2/TNF-α, dual IL-2/TNF-α and single IFN-γ expression in HEU infants at this time point (Fig 2C). Similarly, in CD8 T-cells a significant rise in the magnitude of any cytokine response as well as a trend to a rise in IFN-γ responses was observed in HEU infants at 3 months (Fig 3B); this was mirrored by significant rise in dual IFN-γ/IL-2 and IFN-γ/TNF-α expression and a trend to an rise in single IFN-γ expression at this time point, following polyfunctional analysis (S5B Fig).

At 12 months the opposite observation was made, where, except for IFN-γ, the magnitude of all cytokine responses following SEB stimulation were significantly decreased in HEU infants (Fig 3A); this was mirrored by significant decreases in dual IL-2/TNF-α and single TNF-α responses and a trend towards reduced triple IFN-γ/IL-2/TNF-α and single IL-2 responses (Fig 2D). Altered CD8 T-cell response to SEB were less apparent at 12 months with trends to decreased TNF-α (Fig 3B) and dual IL-2/TNF-α responses (S5B Fig) in HEU infants.

### Altered memory Th1 cytokine responses following stimulation vaccine antigens in HEU relative to HU infants

We determined the memory phenotype of cytokine responsive cells through surface expression of CD45RA and CCR7 following stimulation with vaccine antigens (S2F Fig).

**Fig 4:**
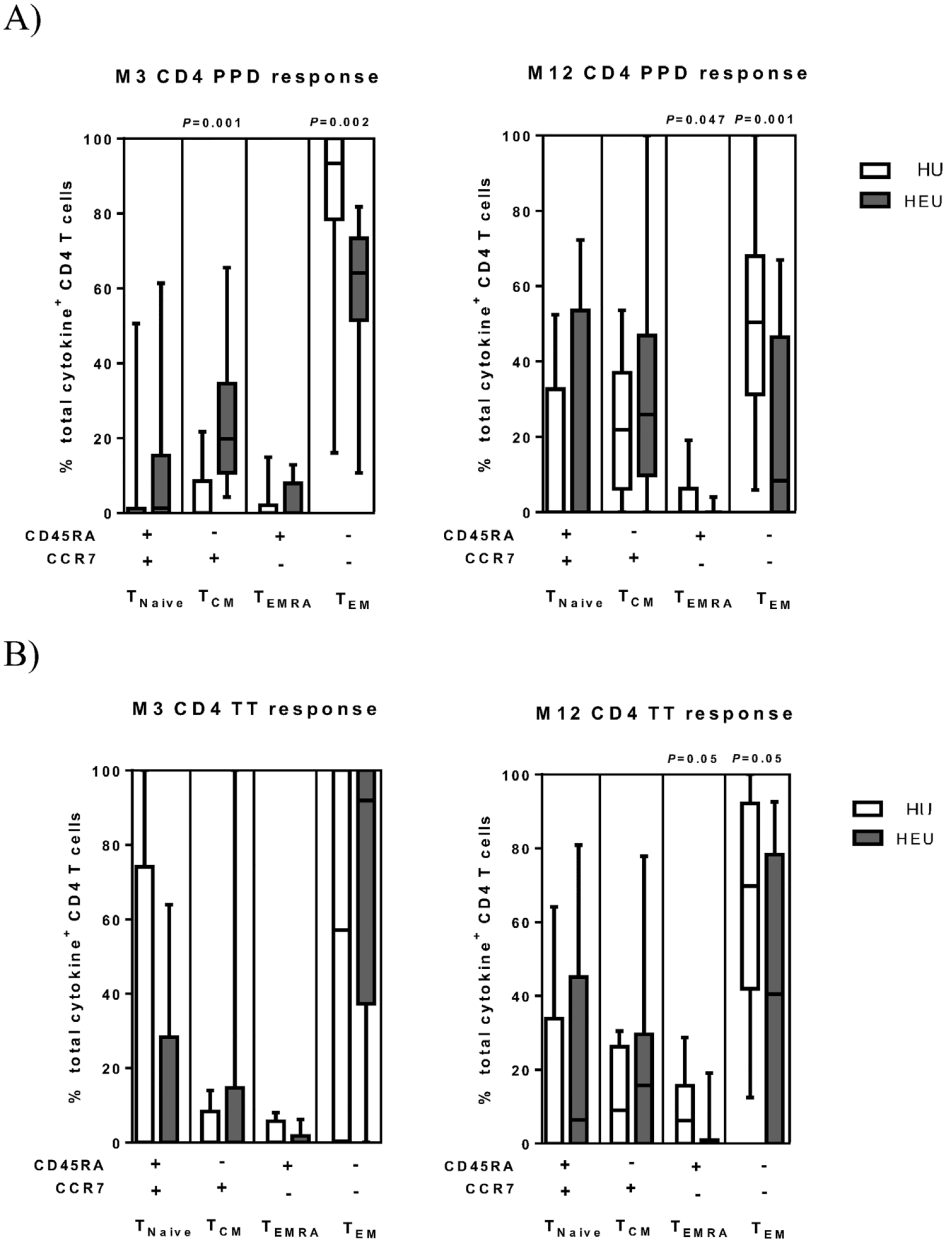
Memory phenotype of vaccine antigen responsive CD4 T-cells. (A) Analysis of the memory phenotype of CD4 T-cells that express any cytokine (IFN-γ, IL-2 or TNF-α) in response to short-term stimulation with PPD. (B) A similar analysis was carried out following short-term stimulation with TT. Comparisons were made between HIV-unexposed (HU) and HIV-exposed uninfected (HEU) infants at month 3 (M3) and month 12 (M12) age groups. The horizontal line in the box and whisker plots is the median percentage of cytokine secreting T-cells with a particular surface memory phenotype, the box is the interquartile range and the whiskers the 10^th^ and 90^th^ percentiles. T_CM_: central memory, T_EM_: effector memory, T_EMRA_: effector memory that re-express CD45RA. The Mann Whitney U test was used to assess differences between the two groups.

Following PPD stimulation we observed a dominant T_EM_ response from CD4 T-cells at 3 months in both HEU and HU infants (Fig 4A). At 12 months, we observed an increase in the proportion of T_CM_ CD4 T-cells contributing to the overall Th1 response. Group comparisons revealed a significant reduction in the proportion of cytokine secreting T_EM_ in HEU infants (Fig 4A) coupled with a significant increase in cytokine secreting T_CM_ cells (Fig 4A). At 12 months, a significant reduction in the proportional response of T_EM_ and T_EMRA_ cells in HEU infants was also observed (Fig 4A). No significant difference were observed in memory T-cell subset frequencies with a positive cytokine response in the CD8 T-cell compartment at either 3 or 12 months (data not shown).

Following TT stimulation, we observed a dominant T_EM_ response from CD4 T-cells at 3 and 12 months in both HEU and HU groups. Group comparisons indicated that at 12 months there was a significant reduction in the proportion of T_EM_ and T_EMRA_ cells that contributed to the overall Th1 response in HEU infants (Fig 4B).

### Immunological findings and maternal characteristics

In order to assess the impact of maternal disease on *ex vivo* immune parameters and antigen specific immune responses a sub analysis was carried out in HEU infants. Maternal HIV viral load at the time of infant recruitment was found to positively correlate with CD8 T cell immune activation at 12 months of age (S6 Fig). By contrast, no association was found between maternal HIV viral load and immune activation in CD4 T cells at any time point or between maternal HIV viral load and the frequency of bulk naïve or T_EM_ cells at 12 months of age in either CD4 or CD8 subsets (S6 Fig).

Analysis of the influence of maternal disease on antigen specific Th1 cytokine responses was assessed by grouping infants according to the maternal CD4 count at the time of recruitment and maternal HAART usage. No significant differences were observed between infants groups in terms of CD4 T cell production of IFN-γ, IL-2, TNF-α or any of these cytokines in response to PPD, TT or SEB stimulation (S4 Table).

## Discussion

A limited understanding exists of factors that mediate poor health in HEU infants particularly regarding immune function. We assessed alterations in the T-cell compartment of HEU infants focusing on *ex vivo* phenotypes and functional responses to the vaccine antigens PPD and TT and polyclonal stimulation. Cross-sectional comparisons were made at 3 and 12 months of age between gender-matched HEU and HU infants vaccinated under the Kenyan Expanded Programme for Immunisation. The influence of maternal health on these parameters was also explored in a sub analysis restricted to HEU infants.

In accordance with recent findings[34], *ex vivo* analyses indicated that immune activation, Treg frequencies and the frequency of memory subsets were closely matched between groups. The exception was reduced activation marker expression on CD4 T-cells at 3 and 12 months and reduced PD-1 expression at 3 months in HEU infants, though activated CD4 T-cell frequencies were typically low (<10% of CD4 T-cells). Reports have shown increased mean fluorescence intensity of CD38 expression on HEU CD4 and CD8 T-cells[13], increased CD38^hi^ CD8 T-cells frequencies[14] and increased PD-1 expression on CD4 T-cells[35] in HEU infants, indicating heightened immune activation and possible exhaustion. We observed, as have others[36], constitutive CD38 expression on infant T-cells; we therefore assessed CD38/HLA-DR co-expression[37]. Our contrasting results with previous reports may be explained through differential exposures to cotrimoxazole which was given to HEU infants prophylactically but not to HU infants. This may have reduced bacterial infections in HEU infants and, coupled with the anti-inflammatory properties of cotrimoxazole [38], had an impact on CD4 T-cell activation and PD-1 expression. However, the low levels of CD4 T cell activation and the similarities in median CD8 T cell activation between groups indicate that factors other than differential cotrimoxazole exposure may play a prominent role in modulation of T cell activation in this setting. Indeed, we observed wide variation in the frequency of activated CD8 T cells in HU and HEU infants at both time points. Our findings, as well as those of others [34] strongly suggest that CD8 T cell activation, in particular, is influenced by maternal HIV viral load. In addition, the role of CMV infection in modulating CD8 T cell immune activation is well documented [39,40] and an increased susceptibility of HEU infants to CMV infection may account for differences in immune activation levels between HEU and HU infants observed in other cohorts. The relationship between maternal HIV viral load and early CMV infection in influencing CD8 T cell activation in HEU infants merits further investigation.

We hypothesised that increased immune activation would influence Treg frequencies. Heightened Treg frequencies have been reported in HEU infants[17], though inconsistently[41]. Our data support an unaltered immune-regulatory potential in HEU infants, as far as can be discerned from circulating Treg frequencies.

Studies on ART-exposed and unexposed HEU infants indicate that naïve T-cells frequencies are reduced in HEU infants[13,14], and this has been interpreted as a mark of *in utero*/early-life priming of naïve T-cells through exposure to HIV-1 or other infectious agents. Our results, and those recently reported in 1 month old Mozambican infants[34], do not indicate alterations in naïve and memory CD4 and CD8 T-cell distributions in HEU infants compared to controls. This supports the notion that early exposure to HIV antigens is reduced in infants born to mothers with ART-mediated virological suppression in turn reducing naïve T-cell priming. In accordance, maternal HIV viral load has been reported to negatively correlate with naïve CD8 T cell frequencies at 1 month of age in HEU infants[34]. Our results did not indicate a similar relationship, probably on account of the small sample size. Alternatively, by 12 months of age, any alterations in bulk memory T-cell subset distributions in HEU infants derived from exposure to maternal HIV may have normalised.

In contrast to the similarities seen *ex vivo* between infant groups and the influence of maternal disease on modulating some these parameters in HEU infants, analysis of T-cell responses following stimulation indicated age-dependent alterations in cytokine production in HEU infants, particularly in CD4 T-cells, that were largely independent of markers of maternal disease. This effect was most striking following SEB stimulation where at 3 months all Th1 cytokine responses were enhanced in HEU infants; conversely, at 12 months IL-2 and TNF-α responses were reduced in HEU infants, despite an overall increase in absolute T cell counts and a trend to increased CD4 T cell counts. This pattern of alterations was also reflected in polyfunctional responses. Notably, reductions in dual IL-2/TNF-α and possibly triple IFN-γ/IL-2/TNF-α expressing cells at 12 months indicate a possible impairment in Th1 functional quality. Longitudinal analyses are needed to assess if the early enhanced Th1 response is associated with the subsequent contraction at 12 months and if these alterations persist. In addition, although no alteration was observed in the magnitude of the 12 month IFN-γ response to SEB in HEU infants, the early life response to SEB matures and increases in magnitude throughout infancy and childhood[42]. Therefore, long-term monitoring of IFN-γ and other cytokine responses into childhood in HEU infants in response to polyclonal stimulation are warranted to assess if the broad protective roles of Th1 cells are impaired.

Responses to vaccine antigens mirrored some of the polyclonal responses despite comparable frequencies of infant responders to vaccination. Following PPD stimulation we observed higher production of any cytokine and dual IL-2/TNF-α production in CD4 T-cells at 3 months in HEU infants. These changes were not apparent at 12 months, suggesting a transient enhancement of immunological reactivity following BCG vaccination in HEU infants in this cohort. Accordingly, increased BCG-specific CD4 and CD8 T-cell proliferation has been reported in 16-week-old HEU infants[43]. By contrast, reduced proliferative responses following BCG stimulation were reported at 10 weeks[35] and at a median of 7 months of age[44] in HEU infants. In addition, Mansoor reported robust mono and polyfunctional BCG-specific cytokine responses in HEU infants throughout the first year of life[45]. We did not assess maternal/infant exposure to *M*. *tuberculosis*, but increased exposure in HEU infants may lead to enhanced early BCG-specific responses. However, a recent study indicated that maternal *M*. *tuberculosis* sensitization had no effect on BCG-specific responses in 16 week-old infants[46]. Alternatively, HEU infants may experience broad hyper-responsiveness to numerous antigens in the first months of life, a hypothesis supported by our observations with SEB stimulation. Additional studies may be needed to define the temporal dynamics of altered responses to BCG in HEU infants.

Following TT stimulation, we observed predominant IL-2 and TNF-α CD4 T-cell responses with minimal frequencies of IFN-γ-responsive cells. No significant alterations in cytokine responses were detected at 3 months in HEU infants; however at 12 months a significant reduction in IL-2 and dual IL-2/TNF-α expressing Th1 cells was observed. T-cell cytokine responses following *in vitro* whole-blood stimulation with TT are reported to result in reduced IFN-γ, IL-5 and IL-13 production in HEU infants at 12 months[47]. Because maternal TT immunisation can prime infant TT-specific responses[48], it was suggested that reduced cytokine secretion in HEU infants results from compromised transplacental transmission of immune complexes consisting of TT and maternal antibodies[47]. Nevertheless, TT vaccination usually induces protective antibody titres in HEU infants, albeit with reduced geometric mean anti-tetanus titres compared to healthy controls[49]. Therefore, it seems that despite compromised mono and dual Th1 and other cytokine response to TT vaccination the protective efficacy, at least in the short-term, of TT is not significantly diminished in HEU infants.

We principally aimed to examine memory CD4 T-cell induction in HEU infants. We observed that following PPD and TT stimulation Th1 responses were mediated predominantly by T_EM_ cells. This finding is supported by reports for BCG[50–52] and TT[28] vaccinations, though not by a recent longitudinal study in South African infants which identified BCG-specific T_CM_ cells as the prominent memory subtype albeit using a distinct anti-CCR7 monoclonal antibody [33]. Crucially, we found that HEU infants had reduced T_EM_ responses following PPD stimulation at 3 and 12 months and at 12 months following TT stimulation. Interestingly, the reduction in antigen-specific T_EM_ responses at 12 months coincided with a trend towards a reduction in the frequency of bulk T_EM_ cells in HEU infants. In addition, following PPD stimulation, the lower T_EM_ response occurred together with a higher T_CM_ response at 3 months. The implications of these observations for protective immunity are not clear, particularly because the immunological correlates of protection for BCG are not defined. In addition, to our knowledge, studies assessing susceptibility patterns to *M*. *tuberculosis* or tetanus are lacking in HEU infants. Therefore our results highlight the need to assess the long-term functional consequences of altered T-cell responses to vaccines in HEU infants.

Our study has a number of strengths and some weaknesses. The former include the comprehensive analysis of *ex vivo* and functional T cell parameters in infants at two key time-points. In addition, the method used to analyse vaccine-specific T cell responses using cryopreserved stimulated leucocytes[26] enabled batch-processing of samples reducing inter-patient experimental error. Finally, to our knowledge, this is the first study to analyse and identify alterations in memory T-cell subset responses to vaccination in HEU infants. Our study was limited principally by our samples size: though a robust cross-sectional analysis could be carried out, meaningful multivariate analyses could not be done. In addition, because the study was not based on a birth cohort, possible confounding factors such gestational age and maternal/infant exposure to *M*. *tuberculosis* and baseline nutritional data for HU infants could not be obtained. Our data should therefore be interpreted with these limitations in mind. Lastly, cotrimoxazole prophylaxis was only provided to HEU infants, in accordance with PMTCT policy in Kenya. The possible immunomodulatory effects of cotrimoxazole may therefore remain a factor that impacts HEU infant immune function. However, the limited data that exists on the effect of cotrimoxazole on T-cell function in humans indicates negligible effects on T cell number[53], proliferation, and IFN-γ, IL-2 or TNF-α secretion following mitogen stimulation[38]. We therefore consider differential cotrimoxazole prophylaxis to have a limited impact on the findings presented here.

In summary, our data add to the body of evidence suggesting specific alterations in T-cell responses to BCG[43,44,54,55] and TT[47] antigens and to polyclonal stimulation[43] in HEU infants. Importantly, we describe alterations in the induction of immunological memory in response to vaccination highlighting the need to monitor long-term outcomes of vaccination in HEU infants and its links to the heightened morbidity and mortality caused by infectious diseases in this vulnerable population.

## Supporting Information

S1 Fig Infant recruitment and specimen inclusion. Flow chart showing the number of infants recruited onto the study in the HIV-unexposed (HU) and HIV-exposed uninfected (HEU) groups, exclusions made due to positive HIV diagnosis and the number of infant specimens included in the analysis at 3 months (M3) and 12 months (M12) of age. *Specimens were obtained from N = 16 HEU infants at both month 3 and 12 months of age.
(DOCX)

S2 Fig Gating strategy for detection of antigen-specific T cells and their memory phenotypes. A) Lymphocytes were gated on based on size and granularity. B) Doubled events were excluded. C) CD3^+^ T cells were gated on and D) the CD4 marker was used to distinguish between CD4 T cells (CD4^+^) and CD8 T cells (CD4^-^). CCR7 is expressed on a clear CD8 T cell population enabling a gate to be placed on the less distinct CD4 T cell populations expressing CCR7. This method was used to aid gate setting for memory phenotypes in F. E) An example plot of ICS for CD4 T cells expressing IFN-γ, IL-2 and TNF-α in unstimulated cells and in cells stimulated with PPD, TT and polyclonally with SEB. A similar approach was used to measure CD8 T cell responses. F) The memory phenotype of cell expressing any cytokine following PPD stimulation is shown overlaid on the memory phenotype of total CD4 T cells. A similar approach was used to determine the memory phenotype of cytokine expressing Th1 cells following TT stimulation. G) T cells with a naïve, effector memory (T_EM_), central memory (T_CM_) and effector memory that re-express CD45RA (T_EMRA_) phenotype can be distinguished in different quadrants as indicated.
(DOCX)

S3 Fig Frequency of infants with a positive cytokine response following stimulation with BCG and TT vaccine antigens. The percentage of HIV-unexposed (HU) control infants (white bars) and HIV exposed-uninfected (HEU) infants (black bars) that produce IFN-γ, IL-2 or TNF-α in response to stimulation with PPD (A) or TT (B) vaccine antigens at 3 and 12 months of age in CD4 and CD8 T cells. *χ* ^*2*^ was used to compare responder frequencies between groups.
(DOCX)

S4 Fig Polyfunctional CD4 T cells responses to stimulation with PPD and TT. (A) CD4 T cells expressing combinations of IFN-γ, IL-2 and or TNF-α were analysed using Boolean gating following short-term stimulation with PPD in HIV-unexposed (HU) and HIV exposed uninfected (HEU) infants in the month 12 (M12) age group. (B) A similar analysis is shown assessing TT responses in the month 3 (M3) age group. The black line is the median frequency of T cells expressing the indicated cytokine combination, the box is the interquartile range and the whiskers the 10^th^ and 90^th^ percentiles. The Mann Whitney U test was used to assess differences between the two groups.
(DOCX)

S5 Fig Polyfunctional CD8 T cells responses to stimulation with PPD and SEB. (A) CD8 T cells expressing combinations of IFN-γ, IL-2 and or TNF-α were analysed using Boolean gating following short-term stimulation with PPD in HIV-unexposed (HU) and HIV exposed uninfected (HEU) infants in the month 3 (M3) and 12 (M12) age group. (B) A similar analysis is shown assessing SEB responses in the M3 and M12 age groups. The black line is the median frequency of T cells expressing the indicated cytokine combination, the box is the interquartile range and the whiskers the 10^th^ and 90^th^ percentiles. The Mann Whitney U test was used to assess differences between the two groups.
(DOCX)

S6 Fig Association between maternal HIV viral load and infant T cell immune activation and memory phenotypes. Associations between maternal viral load and *ex vivo* CD4 and CD8 T cell activation at 3 (n = 16; A) and 12 (n = 13; B) months of age and associations between maternal viral load and infant *ex vivo* CCR7^+^/CD45RA^+^ naïve (C) and CCR7^-^/CD45RA^-^ T_EM_ (D) cell frequencies 12 months only (n = 13). Spearman correlation coefficient (rho) as well as the corresponding *P* value is indicated on each graph.
(DOCX)

S1 Table Antibodies used for immunophenotyping and ICS analysis. (DOCX)

S2 Table Absolute CD3, CD4 and CD8 T cell counts in HU and HEU infants. (DOCX)

S3 Table *Ex vivo* CD8 T cell immune activation and memory cell phenotypes. (DOCX)

S4 Table Influence of maternal CD4 count and ART exposure on infant Th1 responses. (DOCX)
