# Supplementary material for: Altered Memory T-Cell Responses to Bacillus Calmette-Guerin and Tetanus Toxoid Vaccination and Altered Cytokine Responses to Polyclonal Stimulation in HIV-Exposed Uninfected Kenyan Infants
Source: PLoS One. 2015 Nov 16;10(11):e0143043. doi: 10.1371/journal.pone.0143043 (PMC4646342; doi:10.1371/journal.pone.0143043)
Supplement: S1 Fig — Flow chart showing the number of infants recruited onto the study in the HIV-unexposed (HU) and HIV-exposed uninfected (HEU) groups, exclusions made due to positive HIV diagnosis and the number of infant specimens included in the analysis at 3 months (M3) and 12 months (M12) of age. *Specimens were obtained from N = 16 HEU infants at both month 3 and 12 months of age. (DOCX) [file pone.0143043.s001.docx]

Study cohort

N=70

HEU infants

N=42

HU infants

N=28

M3

n=10

M12

n=18

HIV positive

N=5

M3

n=19

M12

n=34

Specimens

N=53*

Specimens

N=28
