# Supplementary figures and images for: Altered Memory T-Cell Responses to Bacillus Calmette-Guerin and Tetanus Toxoid Vaccination and Altered Cytokine Responses to Polyclonal Stimulation in HIV-Exposed Uninfected Kenyan Infants
Source: PLoS One. 2015 Nov 16;10(11):e0143043. doi: 10.1371/journal.pone.0143043 (PMC4646342; doi:10.1371/journal.pone.0143043)

A) B) C) D)


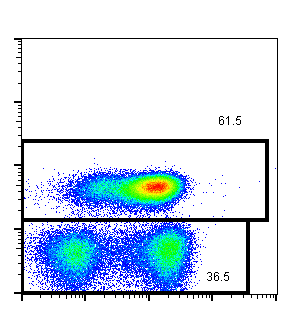


CCR7 APC

CD4 PerCP


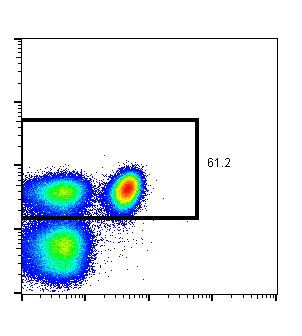


CD3 PB

CD4 PerCP


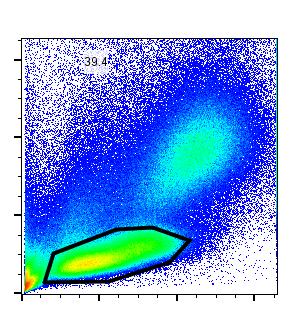


FSC

SSC


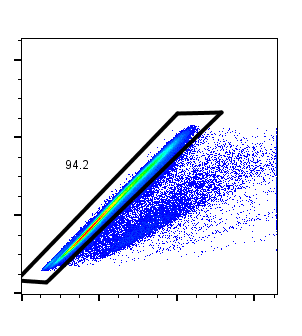


FSC-A

FSC-L

E)

F)
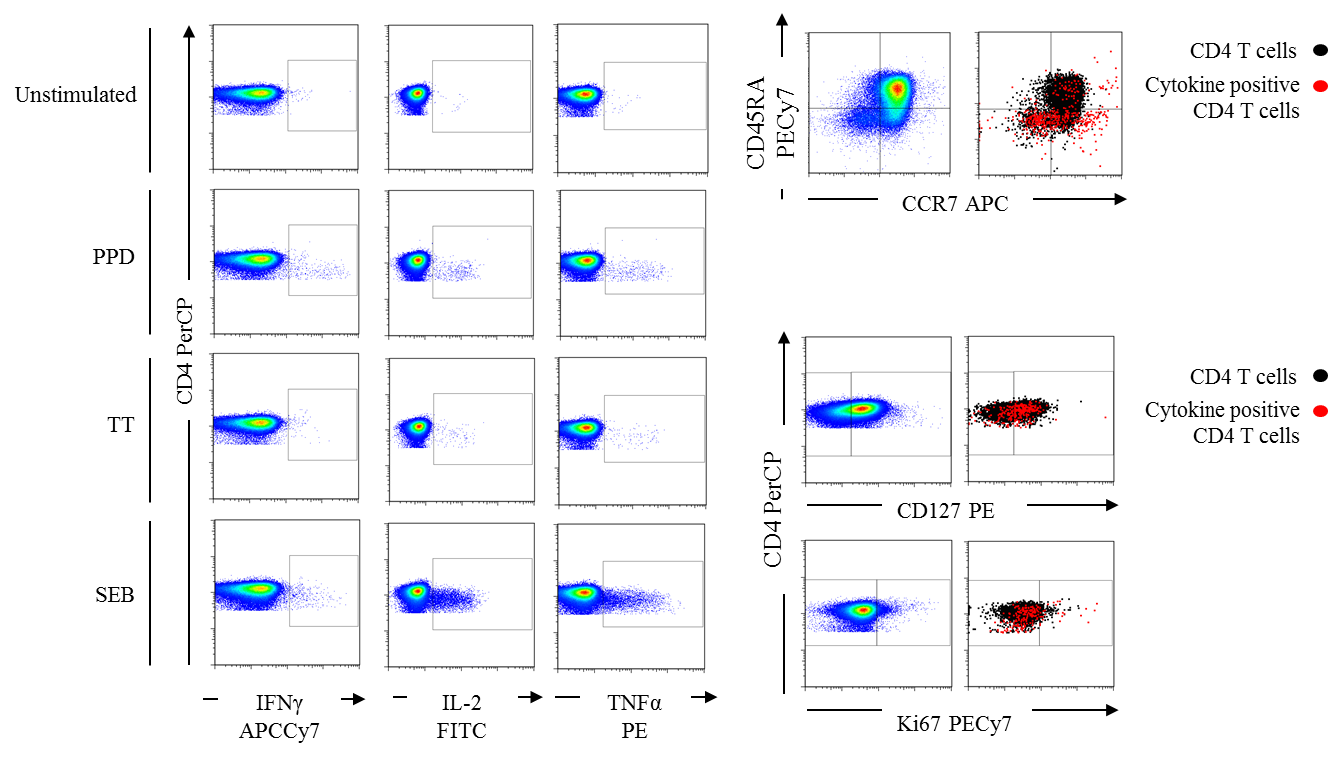
G)
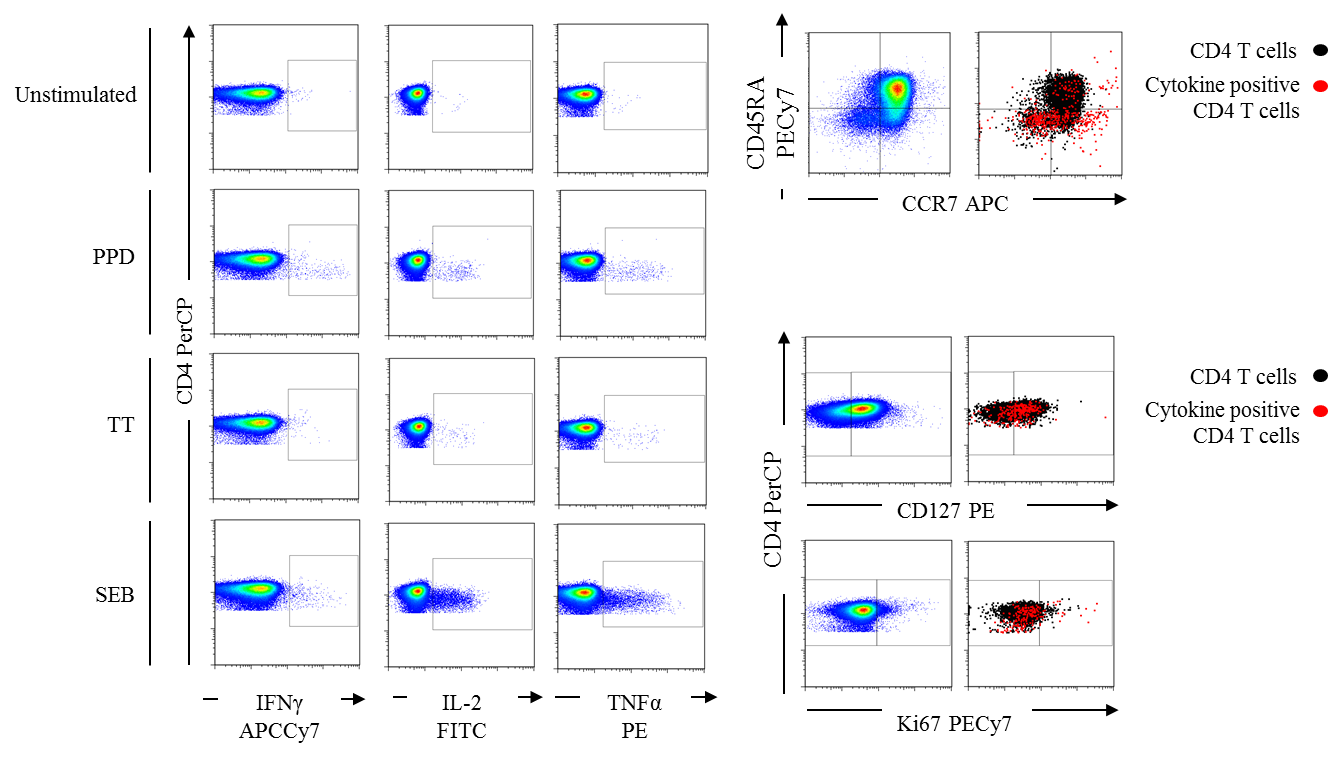


Naive

TEMRA

**TEM**

**TCM**

**CCR7**

**CD45RA**

Supplement: S2 Fig — A) Lymphocytes were gated on based on size and granularity. B) Doubled events were excluded. C) CD3+ T cells were gated on and D) the CD4 marker was used to distinguish between CD4 T cells (CD4+) and CD8 T cells (CD4-). CCR7 is expressed on a clear CD8 T cell population enabling a gate to be placed on the less distinct CD4 T cell populations expressing CCR7. This method was used to aid gate setting for memory phenotypes in F. E) An example plot of ICS for CD4 T cells expressing IFN-γ, IL-2 and TNF-α in unstimulated cells and in cells stimulated with PPD, TT and polyclonally with SEB. A similar approach was used to measure CD8 T cell responses. F) The memory phenotype of cell expressing any cytokine following PPD stimulation is shown overlaid on the memory phenotype of total CD4 T cells. A similar approach was used to determine the memory phenotype of cytokine expressing Th1 cells following TT stimulation. G) T cells with a naïve, effector memory (TEM), central memory (TCM) and effector memory that re-express CD45RA (TEMRA) phenotype can be distinguished in different quadrants as indicated. (DOCX) [file pone.0143043.s002.docx]
