# Supplementary material for: Altered Memory T-Cell Responses to Bacillus Calmette-Guerin and Tetanus Toxoid Vaccination and Altered Cytokine Responses to Polyclonal Stimulation in HIV-Exposed Uninfected Kenyan Infants
Source: PLoS One. 2015 Nov 16;10(11):e0143043. doi: 10.1371/journal.pone.0143043 (PMC4646342; doi:10.1371/journal.pone.0143043)
Supplement: S3 Fig — The percentage of HIV-unexposed (HU) control infants (white bars) and HIV exposed-uninfected (HEU) infants (black bars) that produce IFN-γ, IL-2 or TNF-α in response to stimulation with PPD (A) or TT (B) vaccine antigens at 3 and 12 months of age in CD4 and CD8 T cells. χ 2 was used to compare responder frequencies between groups. (DOCX) [file pone.0143043.s003.docx]

A)

HU

B)

HU
