# Supplementary material for: Altered Memory T-Cell Responses to Bacillus Calmette-Guerin and Tetanus Toxoid Vaccination and Altered Cytokine Responses to Polyclonal Stimulation in HIV-Exposed Uninfected Kenyan Infants
Source: PLoS One. 2015 Nov 16;10(11):e0143043. doi: 10.1371/journal.pone.0143043 (PMC4646342; doi:10.1371/journal.pone.0143043)
Supplement: S4 Fig — (A) CD4 T cells expressing combinations of IFN-γ, IL-2 and or TNF-α were analysed using Boolean gating following short-term stimulation with PPD in HIV-unexposed (HU) and HIV exposed uninfected (HEU) infants in the month 12 (M12) age group. (B) A similar analysis is shown assessing TT responses in the month 3 (M3) age group. The black line is the median frequency of T cells expressing the indicated cytokine combination, the box is the interquartile range and the whiskers the 10th and 90th percentiles. The Mann Whitney U test was used to assess differences between the two groups. (DOCX) [file pone.0143043.s004.docx]

A) B)

HU

HU
