# Supplementary material for: Altered Memory T-Cell Responses to Bacillus Calmette-Guerin and Tetanus Toxoid Vaccination and Altered Cytokine Responses to Polyclonal Stimulation in HIV-Exposed Uninfected Kenyan Infants
Source: PLoS One. 2015 Nov 16;10(11):e0143043. doi: 10.1371/journal.pone.0143043 (PMC4646342; doi:10.1371/journal.pone.0143043)
Supplement: S6 Fig — Associations between maternal viral load and ex vivo CD4 and CD8 T cell activation at 3 (n = 16; A) and 12 (n = 13; B) months of age and associations between maternal viral load and infant ex vivo CCR7+/CD45RA+ naïve (C) and CCR7-/CD45RA- TEM (D) cell frequencies 12 months only (n = 13). Spearman correlation coefficient (rho) as well as the corresponding P value is indicated on each graph. (DOCX) [file pone.0143043.s006.docx]

A)

B)

C)

D)
