# Supplementary material for: Altered Memory T-Cell Responses to Bacillus Calmette-Guerin and Tetanus Toxoid Vaccination and Altered Cytokine Responses to Polyclonal Stimulation in HIV-Exposed Uninfected Kenyan Infants
Source: PLoS One. 2015 Nov 16;10(11):e0143043. doi: 10.1371/journal.pone.0143043 (PMC4646342; doi:10.1371/journal.pone.0143043)
Supplement: S1 Table — (DOCX) [file pone.0143043.s007.docx]

S1 Table. Antibodies used for immunophenotyping and ICS analysis

| **Marker** | **Fluorochrome** | **Clone** | **Location** | **Manufacturer** |
| --- | --- | --- | --- | --- |
| **Activation & exhaustion** |  |  |  |  |
| CD3 | Pacific Blue | SP34-2 | Surface | BD |
| CD4 | PerCP | RPA-T4 | Surface | BioLegend |
| CD8 | APC H7 | SK1 | Surface | BD |
| HLA-DR | FITC | LN3 | Surface | BioLegend |
| CD38 | PE Cy7 | HIT2 | Surface | Biolegend |
| CD279 (PD-1) | APC | eBioJ105 | Surface | eBiosciences |
| Tim-3 | PE | F38-2E1 | Surface | Biolegend |
| **Memory** |  |  |  |  |
| CD3 | Pacific Blue | SP34-2 | Surface | BD |
| CD4 | PerCP | RPA-T4 | Surface | BioLegend |
| CD8 | Qdot 605 | 3B5 | Surface | Invitrogen |
| CD45RA | PE Cy7 | L48 | Surface | BD |
| CCR7 | APC | 150503 | Surface | R&D Systems |
| CD127 (IL-7R) | PE | HIL-7R-M21 | Surface | BD |
| Bcl-2 | FITC | Bcl-2/100 | Intracellular | BD |
| **Regulatory T cells** |  |  |  |  |
| CD3 | APC H7 | SK7 | Surface | BD |
| CD4 | Pacific Blue | RPA-T4 | Surface | BD |
| CD25 | PE | 2A3 | Surface | BD |
| FoxP3 | FITC | PCH101 | Intranuclear | eBiosciences |
| **Vaccine responses by ICS** |  |  |  |  |
| CD3 | Pacific Blue | SP34-2 | Surface | BD |
| CD4 | PerCP | RPA-T4 | Surface | BioLegend |
| CD45RA | PE Cy7 | L48 | Surface | BD |
| CCR7 | APC | 150503 | Surface | R&D Systems |
| IFN-γ | APC Cy7 | 4S.B3 | Intracellular | BioLegend |
| IL-2 | FITC | MQ-17H12 | Intracellular | BioLegend |
| TNF-α | PE | MAb11 | Intracellular | BD |

APC: allophycocyanin; PerCP: peridinin chlorophyll; FITC: fluorescein isothiocyanate;

PE: phycoerythrin. BD Bioscience, San Jose, CA, USA; ebiosiences San Diego, CA, USA; Invitrogen, Carlsbad, CA, USA; Biolegend, San Diego, CA, USA; R&D Systems Minneapolis, MN, USA
