# Supplementary material for: Altered Memory T-Cell Responses to Bacillus Calmette-Guerin and Tetanus Toxoid Vaccination and Altered Cytokine Responses to Polyclonal Stimulation in HIV-Exposed Uninfected Kenyan Infants
Source: PLoS One. 2015 Nov 16;10(11):e0143043. doi: 10.1371/journal.pone.0143043 (PMC4646342; doi:10.1371/journal.pone.0143043)
Supplement: S2 Table — (DOCX) [file pone.0143043.s008.docx]

**S2 Table. Absolute CD3, CD4 and CD8 T cell counts in HU and HEU infants**

| **Parameter** | **M3** | | ****P* value** | **M12** | | ***P* value** |
| --- | --- | --- | --- | --- | --- | --- |
|  | **HU (n=10)** | **HEU (n=19)** |  | **HU (n=16)** | **HEU (n=16)** |  |
| CD3 T cells/µL x10^3^, median (5-95^th^ percentile) | 3.2 (2.5-4.8) | 3.8 (2.9-4.6) | 0.88 | 3.4 (1.4-6.4) | 4.8 (2.8-6.6) | 0.01 |
| CD4 T cells/µL x10^3^, median (5-95^th^ percentile) | 2.1 (1.6-2.6) | 2.2 (1.6-2.7) | 0.98 | 2.2 (0.6-3.9) | 2.6 (1.5-4.2) | 0.08 |
| CD8 T cells/µL x10^3^, median (5-95^th^ percentile) | 0.9 (0.5-1.7) | 0.9 (0.7-1.7) | 0.61 | 1.0 (0.5-2.0) | 1.8 (0.7-2.3) | 0.02 |
| CD4 %, median (5-95^th^ percentile) | 32.0 (26.3-50.2) | 31.4 (7.9- 43.8) | 0.61 | 34.9 (23.2-46.0) | 33.2 (16.7-48.6) | 0.98 |
| CD8 %, median (5-95^th^ percentile) | 15.8 (6.8-34.5) | 19.1 (7.7-35.8) | 0.25 | 19.3 (7.0-30.1) | 19.2 (10.4-35.0) | 0.47 |
| CD4/CD8 T cell ratio (5-95^th^ percentile) | 2.4 (0.9-6.1) | 1.7 (0.3-5.1) | 0.21 | 1.9 (0.9-3.5) | 1.8 (0.7-3.6) | 0.68 |

*M3, month 3; M12, month 12; HU, HIV unexposed; HEU, HIV exposed uninfected. ^¥^ *P* values were calculated using an unpaired T test for normally distributed data or a Mann-Whitney U test for data not normally distributed.
