# Supplementary material for: Altered Memory T-Cell Responses to Bacillus Calmette-Guerin and Tetanus Toxoid Vaccination and Altered Cytokine Responses to Polyclonal Stimulation in HIV-Exposed Uninfected Kenyan Infants
Source: PLoS One. 2015 Nov 16;10(11):e0143043. doi: 10.1371/journal.pone.0143043 (PMC4646342; doi:10.1371/journal.pone.0143043)
Supplement: S3 Table — (DOCX) [file pone.0143043.s009.docx]

**S3 Table. *Ex vivo* CD8 T cell immune activation and memory phenotypes**

| Characteristic | 3 months  Median % of CD8 T cells (range) | | | 12 months  Median % of CD8 T cells (range) | | |
| --- | --- | --- | --- | --- | --- | --- |
|  | **HU (n=10)** | **HEU (n=19)** | **^¥^*P*** | **HU (n=16)** | **^Φ^HEU(n=16)** | **^¥^*P*** |
| Activation & exhaustion |  |  |  |  |  |  |
| CD38^+^ HLA-DR^+^ | 48.6 (2.5-65.2) | 37.8 (3.5-75.4) | NS | 40.6 (14.8-75.5) | 32.0 (10.2-72.9) | NS |
| PD-1 | 28.3 (12.7-47.1) | 22.7 (2.8-47.3) | NS | 32.3 (3.6- 87.2) | 24.6 (9.6-92.3) | NS |
| Tim-3 | 18.4 (9.5-25.9) | 13.1 (2.6-78.1) | NS | 19.4 (4.8-62.4) | 23.2 (9.6-69.2) | NS |
| Anti-apoptosis |  |  |  |  |  |  |
| Bcl-2^+^ | - | - | NA | 58.8 (26.5-96.4) | 60.7 (27.4-93.1) | NS |
| Bcl-2^-^ | - | - | NA | 41.3 (3.6-73.5) | 29.4 (6.4-72.6) | NS |
| Memory |  |  |  |  |  |  |
| CD127^+^ | - | - | NA | 49.3 (26.4-92.9) | 45.5 (18.1-67.6) | NS |
| CD127^-^ | - | - | NA | 51.1 (7.12-73.6) | 54.5 (32.0-81.9) | NS |
| Naïve | - |  | NA | 49.1 (25.6-86.8) | 58.9 (21.0-97.1) | NS |
| T_CM_ | - | - | NA | 2.9 (0.7-9.5) | 2.0 (0.3-7.7) | NS |
| T_EMRA_ | - | - | NA | 30.0 (2.98-65.0) | 27.4 (0.7-63.3) | NS |
| T_EM_ | - | - | NA | 13.5 (2.9-32.0) | 9.6 (0.2-46.0) | NS |

^Φ^CD127 expression was measured on n=11 samples ^¥^ *P* values were calculated using the Mann-Whitney U test.

NS=not significant NA= not applicable; HEU, HIV exposed uninfected; HU, HIV unexposed
