# Supplementary material for: Altered Memory T-Cell Responses to Bacillus Calmette-Guerin and Tetanus Toxoid Vaccination and Altered Cytokine Responses to Polyclonal Stimulation in HIV-Exposed Uninfected Kenyan Infants
Source: PLoS One. 2015 Nov 16;10(11):e0143043. doi: 10.1371/journal.pone.0143043 (PMC4646342; doi:10.1371/journal.pone.0143043)
Supplement: S4 Table — (DOCX) [file pone.0143043.s010.docx]

**S4 Table. Influence of maternal CD4 count and ART exposure on infant Th1 responses**

| Maternal characteristic | | Infant Th1 response | | | | | | | |
| --- | --- | --- | --- | --- | --- | --- | --- | --- | --- |
|  |  | M3 PPD response, median frequency of CD4 T cells (IQR) | | | | | | | |
|  |  | IFN-γ | *P* | IL-2 | *P* | TNF-α | *P* | Any | *P^¥^* |
| CD4 count* | >350 (n=7)  <350 (n=4) | 0.05 (0-0.09)  0.09 (0-0.3) | 1.0 | 0.06 (0.04-0.43)  0.09 (0.05-0.38) | 0.8 | 0.11 (0.07-0.49)  0.16 (0.06-0.49) | 0.8 | 0.10 (0.08-0.46)  0.17 (0.07-0.57) | 0.8 |
| HAART exposure | On HAART  (n=6)  No HAART^Φ^ (n=5) | 0.05 (0-0.20)  0.03 (0.01-0.38) | 0.6 | 0.09 (0.05-0.26)  0.06 (0.02-0.55) | 0.7 | 0.10 (0.07-0.32)  0.10 (0.04-0.69) | 0.6 | 0.17 (0.08-0.37)  0.08 (0.04-0.78) | 0.6 |
|  | | M12 PPD response, median frequency of CD4 T cells (IQR) | | | | | | | |
|  |  | IFN-γ | *P* | IL-2 | *P* | TNF-α | *P* | Any | *P* |
| CD4 count | >350 (n=14)  <350 (n=8) | 0.01 (0-0.03)  0.05 (0-0.07) | 0.08 | 0.05 (0.02-0.12)  0.09 (0-0.14) | 0.4 | 0.04 (0.03-0.16)  0.12 (0-0.22) | 0.7 | 0.08 (0.02-0.22)  0.11 (0.03-0.22) | 0.7 |
| HAART exposure | On HAART (n=13)  No HAART  (n=10) | 0.03 (0-0.07)  0.01 (0-0.05) | 0.3 | 0.07 (0.02-0.11)  0.06 (0-0.14) | 1.0 | 0.05 (0.03-0.19)  0.04 (0-0.16) | 0.4 | 0.10 (0.02-0.25)  0.07 (0-0.22) | 0.4 |
|  | | M12 TT response, median frequency of CD4 T cells (IQR) | | | | | | | |
|  |  | IFN-γ | *P* | IL-2 | *P* | TNF-α | *P* | Any | *P* |
| CD4 count | >350 (n=12)  <350 (n=8) | 0 (0-0.01)  0 (0-0.02) | 0.9 | 0.09 (0.03-0.10)  0.06 (0.03-0.11) | 0.7 | 0.04 (0.01-0.12)  0.05 (0.01-0.10) | 0.8 | 0.09 (0.04-0.15)  0.07 (0.04-0.11) | 0.6 |
| HAART exposure | On HAART (n=14)  No HAART (n=7) | 0 (0-0.01)  0.01 (0-0.02) | 0.2 | 0.04 (0.02-0.10)  0.09 (0.06-0.11) | 0.09 | 0.03 (0-0.11)  0.05 (0.03-0.13) | 0.5 | 0.05 (0.03-0.13)  0.10 (0.07-0.14) | 0.1 |
|  | | M3 SEB response, median frequency of CD4 T cells (IQR) | | | | | | | |
|  |  | IFN-γ | *P* | IL-2 | *P* | TNF-α | *P* | Any | *P* |
| CD4 count | >350 (n=8)  <350 (n=4) | 0.15 (0.03-0.25)  0.15 (0.11-0.20) | 0.8 | 1.49 (0.35-3.44)  1.60 (0.95- 1.80) | 0.7 | 0.92 (0.36-2.35)  1.33 (0.88-1.80) | 0.7 | 1.80 (0.53-4.07)  2.15 (1.24-2.53) | 0.7 |
| HAART exposure | On HAART (n=7)  No HAART  (n=5) | 0.15 (0.07-0.20)  0.15 (0.07-0.45) | 0.3 | 1.56 (1.32-1.85)  1.59 (0.59-4.46) | 0.3 | 1.07 (0.71-1.60)  1.04 (0.67-5.15) | 0.2 | 1.98 (1.55-2.60)  1.98 (0.87-6.24) | 0.3 |
|  | | M12 SEB response, median frequency of CD4 T cells (IQR) | | | | | | | |
|  |  | IFN-γ | *P* | IL-2 | *P* | TNF-α | *P* | Any | *P* |
| CD4 count | >350 (n=16)  <350 (n=9) | 0.10 (0.06-0.14)  0.12 (0.10-0.33) | 0.2 | 0.81 (0.39-1.13)  0.08 (0.66-2.00) | 0.2 | 0.65 (0.33-1.33)  1.10 (0.46-1.81) | 0.2 | 1.08 (0.48-1.76)  1.33 (0.78-2.53) | 0.2 |
| HAART exposure | On HAART (n=15)  No HAART (n=11) | 0.09 (0.04-0.12)  0.14 (0.09-0.20) | 0.3 | 0.76 (0.46-1.06)  1.03 (0.53-1.67) | 0.3 | 0.64 (0.37-1.11)  0.94 (0.44-1.60) | 0.2 | 1.06 (0.64-1.34)  1.34 (0.67-2.46) | 0.3 |

*CD4 count at the time of infant recruitment, cells/mm^3^. ^Φ^Mothers not on HAART received prophylactic antiretroviral therapy azidothymidine (AZT) during, through and after delivery, as per the Kenyan guidelines at the time of the study. *^¥^* *P* values were calculated using an unpaired T test for normally distributed data or a Mann-Whitney U test for data not normally distributed. HAART, highly active antiretroviral therapy; IQR, interquartile range. M3, 3 months, M12, 12 months;
